# Short-term retention of relational memory in amnesia revisited: accurate performance depends on hippocampal integrity

**DOI:** 10.3389/fnhum.2014.00016

**Published:** 2014-01-24

**Authors:** Lydia T. S. Yee, Deborah E. Hannula, Daniel Tranel, Neal J. Cohen

**Affiliations:** ^1^Faculty of Medicine, Institute of Cognitive Neurology and Dementia Research, Otto-von-Guericke University MagdeburgMagdeburg, Germany; ^2^Department of Psychology, University of Wisconsin–MilwaukeeMilwaukee, WI, USA; ^3^Departments of Neurology and Psychology, University of IowaIowa City, IA, USA; ^4^Department of Psychology and Beckman Institute for Advanced Science and Technology, University of IllinoisUrbana-Champaign, IL, USA

**Keywords:** amnesia, hippocampus, short-term memory, relational memory, delayed recognition

## Abstract

Traditionally, it has been proposed that the hippocampus and adjacent medial temporal lobe cortical structures are selectively critical for long-term declarative memory, which entails memory for inter-item and item-context relationships. Whether the hippocampus might also contribute to short-term retention of relational memory representations has remained controversial. In two experiments, we revisit this question by testing memory for relationships among items embedded in scenes using a standard working memory trial structure in which a sample stimulus is followed by a brief delay and the corresponding test stimulus. In each experimental block, eight trials using different exemplars of the same scene were presented. The exemplars contained the same items but with different spatial relationships among them. By repeating the pictures across trials, any potential contributions of item or scene memory to performance were minimized, and relational memory could be assessed more directly than has been done previously. When test displays were presented, participants indicated whether any of the item-location relationships had changed. Then, regardless of their responses (and whether any item did change its location), participants indicated on a forced-choice test, which item might have moved, guessing if necessary. Amnesic patients were impaired on the change detection test, and were frequently unable to specify the change after having reported correctly that a change had taken place. Comparison participants, by contrast, frequently identified the change even when they failed to report the mismatch, an outcome that speaks to the sensitivity of the change specification measure. These results confirm past reports of hippocampal contributions to short-term retention of relational memory representations, and suggest that the role of the hippocampus in memory has more to do with relational memory requirements than the length of a retention interval.

Recent studies have indicated that the reach of the hippocampus extends beyond long-term declarative memory to the domain of active, online retention of memory for inter-item and item-context relationships (e.g., Hannula et al., 2006; Olson et al., 2006; Hartley et al., 2007; Finke et al., 2008, 2013; Braun et al., 2011; Watson et al., 2013). These findings are notable because traditionally it has been proposed, and most investigators have agreed, that the hippocampus and adjacent medial temporal lobe (MTL) cortical structures are selectively critical for long-term memory. Such claims were consistent with early reports in the literature that had shown intact short-term retention of digits, tones, estimated number of dots in a display, and single spatial locations following MTL damage (e.g., Sidman et al., 1968; Wickelgren, 1968; Warrington and Baddeley, 1974; Cave and Squire, 1992). Indeed, the stark differences in performances of amnesic patients on short- and long-term memory tasks have often been cited as lynchpin evidence for distinct short- and long-term memory systems in the brain (see Ranganath and Blumenfeld, 2005; Jeneson and Squire, 2011 for review).

We have proposed (cf. Hannula et al., 2006), as have others (e.g., Olson et al., 2006), that the tasks in which intact short-term memory performances have been documented may not have required representation of relationships among items which, by some accounts, depends critically on the integrity of the hippocampus (e.g., Cohen and Eichenbaum, 1993; Davachi, 2006; Eichenbaum et al., 2007; Mayes et al., 2007; Olsen et al., 2012). As indicated above, recent studies that have tested this hypothesis suggest that short-term retention of relational memory representations is compromised following MTL (and especially hippocampal) damage (e.g., Hannula et al., 2006; Olson et al., 2006; Hartley et al., 2007; Finke et al., 2008, 2013; Watson et al., 2013). Notably though, the impaired short-term memory performances that have been documented in these studies sometimes pale in comparison to the devastating impairments on corresponding tests of long-term memory; this is especially apparent when the same materials and testing procedures are used to assess memory at short and long lags (as in Hannula et al., 2006; but see Watson et al., 2013). In addition, the recently reported contributions of MTL structures to short-term or working memory have been questioned (cf. Jeneson and Squire, 2011) and some new findings from other groups have challenged the reported outcomes (e.g., Shrager et al., 2008; Jeneson et al., 2010, 2011; see also Baddeley et al., 2010, 2011). Here, we address criticisms that have been levied against our own previous work, in which participants were required to identify changes in relationships among items embedded in the context of rendered scenes (e.g., a bedroom scene, a kitchen scene; Hannula et al., 2006).

In the above-referenced experiment (Hannula et al., 2006; experiment 1), memory for spatial relationships among items embedded in scenes was tested using a continuous recognition design. Test trials were either presented immediately after corresponding study trials, five trials later, or nine trials later in a continuous sequence. The experiment was designed so that, in the absence of memory for previous exposure to a scene, study trials could not easily be distinguished from test trials. This was because the sequence of trial events was always the same—while a scene was in view, whether it was being presented for the first time or had been seen previously, participants answered three questions. The first question, an “orienting” question, encouraged participants to attend to the location of a critical item (i.e., to ensure processing of the item that might undergo, or might have undergone, a relational change); the remaining two questions assessed memory for scenes (i.e., whether a scene was old or new) and memory for spatial relationships among items embedded in scenes [i.e., whether one of the objects had changed locations; see also Ryan et al. (2000)]. Despite successful discrimination of studied from novel scenes, amnesic patients with hippocampal damage were impaired on the test of relational memory, and this impairment was evident even when test trials were presented immediately after corresponding study trials (i.e., short lag condition). Based on this outcome, we proposed that the role of the hippocampus in memory has more to do with whether or not relational memory representations are required for successful task performance than with the length of the retention interval, a conclusion that was also drawn by Olson et al. (2006) using a different experimental paradigm.

The results reported by Hannula et al. (2006) were replicated subsequently by a different lab using the same task and procedures (Jeneson et al., 2011; see also Jeneson and Squire, 2011), but the conclusion was different. Here, it was proposed that the short-lag impairment was a consequence of memory load that was large enough to exceed the capacity of working memory, and two compelling criticisms of the original work were outlined. The first criticism concerned the length of the delay imposed between trials. As indicated above, scenes were occasionally presented again immediately after they were studied (i.e., lag 1 condition), and here the time separating one scene from the next was just 3 s—a delay commensurate with those used in standard working memory tasks. However, this “delay” does not take into account the actual passage of time between the offset of a “studied” scene and the assessment of memory, which took place subsequent to free viewing of the “test” scene (5 s) and after presentation of the associated orienting question (6 s), which was shown at the bottom of the screen while the “test” scene remained in view. When the timing of these events is considered together, the lag 1 delay (i.e., 14 s) was much longer than the 3 s intertrial interval, and performance may have consequently drawn on long-term memory. The second, and perhaps more problematic criticism, concerns the use of a continuous recognition design. While ideal for assessing memory at short- and long-lags simultaneously, this design choice may have discouraged participants from attempting to actively retain information in memory from one trial to the next. Indeed, even when lag 1 trials were administered, participants may have had to try to retain several other scenes (as many as 9) from earlier, as yet untested, trials. Stated this way, the memory burden on a given trial, even in the lag 1 condition, was potentially quite substantial, and this may have driven the reported impairment.

Because of these concerns, Jeneson et al. (2011) conducted a new version of the experiment with a trial structure that is more commonly used in short-term or working memory tasks. On every trial, a sample stimulus (a scene), was followed by a brief (3 s or 14 s) unfilled delay, and a test stimulus. The test stimulus was either an exact match of the studied scene or a version of that scene in which one item had been moved to a new location. Under these circumstances, amnesic patients were only impaired on the match/mismatch decision when 14 s separated the sample stimulus from the test display. A small, statistically unreliable, group difference following the 3 s delay was driven by the performance of one patient. Based on this outcome, it was concluded that short-term memory for relationships among items in scenes does not depend on the integrity of the hippocampus, a proposal consistent with traditional views of MTL function (cf. Scoville and Milner, 1957).

Two points are worth noting with respect to preserved performance of amnesic patients in this task. First, the control group performed near ceiling (97% correct), which may have precluded identification of any small impairment that would otherwise be evident. Second, the orienting questions, used in the original continuous recognition experiment, were also used in the new study to direct attention to the critical object during the sample phase of each trial. As indicated above, the orienting question was meant to ensure that participants attended to and processed the item that might undergo a relational change, but use of questions like this in a standard short-term memory task is not typical, and may have influenced the findings. For example, in the absence of a lag-based (or continuous recognition) design, participants may have come to rely more on the orienting question than they would have otherwise done, rehearsing this question actively over the course of the delay. In this case, performance on the relational memory test may have been augmented by verbal rehearsal of the question. In addition, it is possible that participants discerned that the object referenced in the orienting question was the only object that would change locations, and that this insight led to active maintenance of the absolute spatial location of that item (e.g., in an egocentric reference frame), which may not depend critically on hippocampal integrity (cf. Burgess, 2008). In other words, participants could have performed well even if they had neglected (or failed to represent) the position of the critical item relative to other items embedded in the scene. As such, it is possible that if we require participants to rely more heavily on inter-item relationships, and discourage verbal rehearsal that may occur when orienting questions are presented, impaired performance will be evident after all. This possibility was examined in the current set of experiments.

The studies that follow examined whether or not the performance of amnesic patients on a test of memory for relationships among items embedded in scenes might be impaired when a more traditional short-term memory testing procedure was used, but orienting questions were omitted. As in Jeneson et al. (2011) each trial in the reported experiments began with a sample display, and relational memory was tested following a brief delay. In the reported studies, and in contrast to the methods used previously, several exemplars of the same scene (e.g., a beach scene) were presented repeatedly across trials as the memoranda; different exemplars were distinguishable because the locations of a subset of items changed from one trial to the next in an experimental block. Repeated use of the same scene (e.g., the beach scene), which itself contained the same items, across a set of trials meant that accurate performance hinged on memory for the spatial relationships among items in the current scene variant. Memory for the items themselves, or for the scene context, would be insufficient to support accurate performance on this short-term memory task. This methodological choice meant that we were able to examine relational memory in relative isolation, free from the influence of item or scene memory, and because scene exemplars were very similar to each other, precise relational memory representations would be required for accurate performance. It was expected that amnesic patients with hippocampal damage would be impaired, even in the absence of a continuous recognition design, and even when a standard short-delay trial structure was adopted. Use of the same scenes/items across a set of trials also meant that we could examine whether or not there were any systematic influences of long-term memory (beneficial, due to improvements in item or scene memory, or detrimental, due to increasing interference across repetitions) that affected the performance of the comparison group.

## Experiment 1

### Methods

#### Participants

Participants were five patients (three male) with amnesia and eight neurologically intact individuals each matched to one of the patients in terms of age, gender, handedness, and education. Patients were drawn from a registry established and maintained by the Division of Neuroscience at the University of Iowa, and comparison participants were recruited from the Champaign-Urbana community. Experimental procedures were approved by the ethics committees at the Universities of Iowa and Illinois, and informed consent was obtained from each participant prior to conducting the study.

In each case, and as described in more detail elsewhere (Buchanan et al., 2005; Allen et al., 2006; Hannula et al., 2006; Warren et al., 2012a,b; Watson et al., 2013), amnesia was secondary to an anoxic event and structural MRI scans, obtained from four patients, confirmed bilateral hippocampal volume reductions. Significant loss was also evident for a subset of these individuals in the parahippocampal gyrus, but these reductions were less extensive than corresponding volume changes in the hippocampus. A coronal MRI scan through the hippocampus for patient 1606, which shows hippocampal volume changes bilaterally can be seen in Bechara et al. (1995), and high-resolution structural MRI scans for patient 1846 can been seen in Warren et al. (2012b). The remaining patient (2563) is not eligible for MRI scanning, but visual inspection of CT scans suggests focal hippocampal damage.

Outside of the MTL, and especially important here given our interest in the integrity of short-term memory, three of four patients had intact frontal and parietal lobe volumes. The remaining patient (2363) had significantly reduced gray matter volume in the parietal lobe, but frontal lobe volume was within normal limits. Studentized residuals, estimates of brain volume integrity relative to a healthy matched comparison group, are provided in Table 1 for each patient (with the exception of 2563) and for all of the regions of interest referenced above (see Allen et al., 2006 for more detail about how these estimates were obtained).

**Table 1:** **Basic demographic information and brain volumes**.

**Patient**	**Age**	**Yrs Ed**	**Hand**	**Sex**	**HC**	**PHG**		**Frontal**		**Parietal**		**Cerebrum**	
						**gray**	**white**	**gray**	**white**	**gray**	**white**	**gray**	**white**
1606	59	12	R	M	−3.99	−2.46	−2.36	−0.66	−0.28	−0.59	−0.73	−1.13	−0.85
1846	43	14	R	F	−4.23	−1.28	−2.19	−1.42	−1.07	−1.79	1.27	−1.54	−1.01
2144	57	12	R	F	−3.92	−1.22	0.65	−1.88	−0.45	−1.11	−0.49	−1.29	−0.37
2363	50	16	R	M	−2.64	−2.26	−0.37	−1.92	1.01	−2.78	−1.17	−2.47	0.07
2563	51	16	L	M	N/A	N/A	N/A	N/A	N/A	N/A	N/A	N/A	N/A

Yrs Ed, Years of Education; HC, Hippocampus; PHG, Parahippocampal Gyrus. Measures of brain volume are studentized residuals calculated relative to brain volumes obtained from a matched healthy comparison group (see Allen et al., 2006 for details). The cut-off for statistically reliable volume reduction was a studentized residual of −2.00 (p < 0.05).

Performances on a standard battery of neuropsychological tests confirmed that each patient had a selective memory impairment that was disproportionate to any small decline in general cognitive or intellectual function. For each patient, performance on the General Memory Index (Wechsler Memory Scale-III) was at least 25 points below their Full-Scale IQ score (Wechsler Adult Intelligence Scale-III), and no more than 11 points (of 36 possible) were obtained when participants attempted to draw the Rey–Osterrieth figure from memory following a delay. In contrast, performances on several standardized tests of working memory were generally within normal limits, a result consistent with empirical studies that have documented intact performances of MTL amnesic patients on STM tasks when memory for simple items was tested (e.g., Cave and Squire, 1992). Scores from several subtests sensitive to working memory are provided in Table 2, among them, the digit span task, an arithmetic task, a sentence repetition task, and a letter-number sequencing task. The arithmetic subtest consists of increasingly complex arithmetic problems that must be solved without the aid of pencil and paper and within a prescribed amount of time. Sentence repetition requires spoken repetition of sentences that are read by an examiner and increase in length from one trial to the next. Letter-number sequencing begins with the examiner reading a series of letters and numbers aloud; examinees then attempt to recall the numbers in ascending order and the letters in alphabetical order. Together, scores from the digit span, arithmetic, and letter-number sequencing subtests yield a composite working memory score (i.e., the Working Memory Index). Scores from all of the standardized neuropsychological tests referenced above are provided for each patient in Table 2.

**Table 2:** **Neuropsychological test scores**.

**Patient**	**WAIS-III**			**WAIS-III (Working memory)**					**WMS-III**	
	**VIQ**	**PIQ**	**FSIQ**	**WMI**	**Digit span**	**Arithmetic**	**LNS**	**Sent Rep**	**GMI**	**CFT**
1606	94	89	91	80	7	9	4	8	66	11
1846	89	79	84	90	10	7	8	11	57	6
2144	102	94	99	95	11	9	8	12	56	3
2363	112	83	98	92	8	11	7	10	73	5
2563	91	105	102	99	14	6	10	13	75	7

WAIS-III, Wechsler Adult Intelligence Scale-III; VIQ, Verbal Intelligence Quotient; PIQ, Performance Intelligence Quotient; FSIQ, Full-Scale Intelligence Quotient; WMI, Working Memory Index; LNS, Letter-Number Sequencing; Sent Rep, Sentence Repetition; WMS-III, Wechsler Memory Scale-III; GMI, General Memory Index; CFT, Complex Figure Task. The VIQ, PIQ, FSIQ, WMI, and GMI yield mean scores in the normal population of 100 with a standard deviation of 15. Digit span, arithmetic, and letter-number sequencing are subtests of the WMI; these are scaled scores with a mean in the normal population of 10 and a standard deviation of 3. Maximum scores for Sent Rep and the CFT are 14 and 36, respectively.

#### Materials

**Figure 1:**
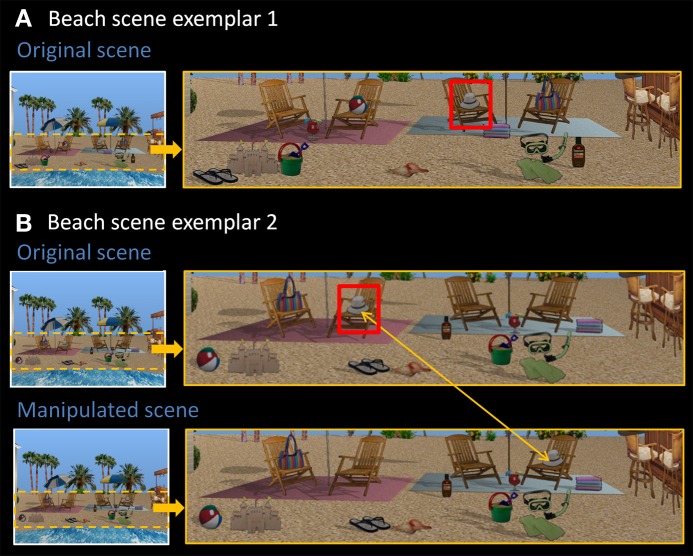
**Stimuli used in Experiment 1. (A)** Exemplar 1 of the beach scene. **(B)** Exemplar 2 of the beach scene, along with its manipulated version. Comparing original exemplars 1 and 2, all eight critical items (sandals, sand bucket, margarita drink, beach ball, hat, towels, bag, sunscreen) have been rearranged into a different spatial configuration. The hat, for example, is now located on the second chair from the left. Red boxes are for illustrative purposes only. In the manipulated version, the critical item (i.e., the hat) has moved from the chair on the left side of the scene to the chair on the right side of the scene, constituting a change in spatial relationships.

Eight rendered scenes (e.g., garage, beach), sized to 960 × 715 pixels, were created using Punch! Home Design Software^©^ (Kansas City, MO). Eight of the objects embedded in each scene were designated “critical items,” and eight distinct exemplars of these scenes were created by changing the locations of these items within the scene context. In other words, the spatial configuration of critical items changed from one exemplar to the next (see Figure 1 for two representative beach scene exemplars). There were four possible locations for each critical item in a given scene context, and each location was filled (or remained empty) equally often. To make the task more difficult, the same location could be occupied by different critical items across scene exemplars. This is illustrated in Figure 1—the beach ball and the flip-flops occupy the same location (both to the left of the sand castle).

There were two different versions of each individual scene exemplar—the original version, and a manipulated version in which one of the eight critical items was moved to a new, albeit equally plausible spatial location, changing spatial relationships among scene elements (see Figure 1B). Each critical item was the target of displacement for exactly one scene exemplar and the new location was always on the opposite side of the scene with left-to-right and right-to-left location changes occurring equally often across exemplars. All together, the final set of materials included 128 scenes—eight distinct scene contexts (e.g., garage, beach), each with eight different exemplars, and one additional manipulated version of each exemplar.

#### Design and procedure

**Figure 2:**
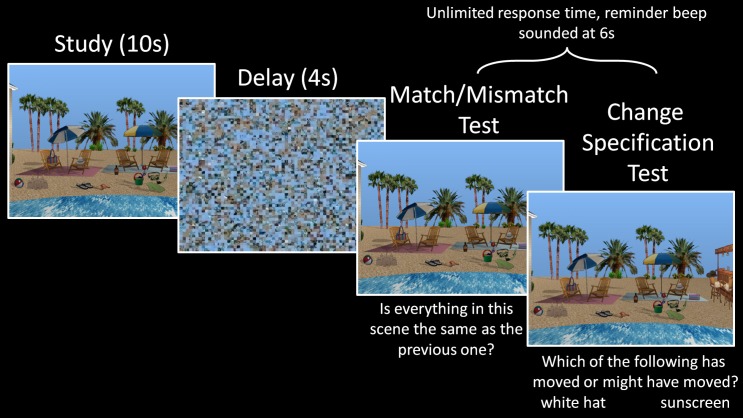
**Trial structure of Experiment 1**. A scene exemplar was studied for 10 s during the sample phase, followed by a 4 s delay, and then a probe phase, where either the same scene exemplar or its manipulated version appeared. During the probe phase, participants first indicated whether the probe was an exact match to the sample scene they had just studied, and then attempted to identify the object that had changed locations (or might have changed locations, in cases where they thought the probe was a match).

The experimental task, which consisted of eight blocks, commenced after instructions were provided and a practice block had been completed; each block, including the practice block, was eight trials in length. All eight exemplars of the same scene (e.g., the beach scene in Figure 1) were studied and tested within the same block, and individual trials were divided into three parts: a sample phase, a short delay, and a probe phase (see Figure 2). Each trial began with the presentation of a scene for 10 s followed by a 4 s delay period. During the delay a scrambled version of the scene, meant to disrupt any additional processing associated with visual persistence of the stimulus, was presented. This visual mask was created by dividing the scene into 195 parts of equal size and then randomly rearranging them into a meaningless configuration. Finally, during the probe phase, participants were shown a scene that was either an exact *match* of the previously studied scene or a *mismatch*, in which one of the critical items was moved to a different spatial location. This scene was presented along with two questions. The first was a global match/mismatch (or change detection) question, which prompted participants to indicate whether the test picture was the same as the one that they had just studied or was a manipulated version of that picture (i.e., “Is everything in this scene the same as the previous one?”). Participants were given unlimited time to respond, but a tone was presented 6 s after probe onset if the behavioral response had not yet been made. Following the first button press, the initial question was replaced with a second question that required participants to identify the item that had changed locations (i.e., “Which of the following has moved or might have moved?”). The names of two critical objects, each present in the scene (which remained in view), were presented at the bottom of the screen and participants made their button press response. Importantly, participants were instructed to select one of the alternatives whether they had indicated the scene was a *match* or a *mismatch*—i.e., even if they felt nothing had changed between study and test, participants were to make a “best guess” as to what might have changed. This forced-choice procedure provided a sensitive measure of memory for spatial relationships among scene elements even when participants adopted a conservative response criterion (i.e., reporting no change when they suspected, but were not certain that a change had taken place). Only the names of critical items that had not undergone a position change in previous trials were used as alternatives, which meant that each participant received different alternatives for the same scene exemplar according to his or her particular viewing history.

Rest was provided in between experimental blocks as needed, and instructions were reiterated before each block was initiated. Three of the patients (2363, 2563, and 1846) completed the experiment twice, with different counterbalanced versions of the task. This was done to achieve full counterbalancing of the scene stimuli (i.e., there were just 5 available patients, but the design was counterbalanced across 8 participants). As is common when patients are tested more than once in neuropsychological investigations, several months separated one session from the next to minimize any contributions of long-term memory to performance. Analyses were based on the averaged data from both sessions for those individuals who completed two rounds of testing, and debriefing was provided at the conclusion of the experiment.

Counterbalancing ensured that individual scene exemplars were presented equally often in both experimental conditions (i.e., match, mismatch) and in the first and second half of an experimental block. Furthermore, each set of scenes (i.e., the beach scenes) was presented equally often across blocks (i.e., block 1, block 2 … block 8). The original scene exemplar and the manipulated version of that exemplar were presented equally often during the sample phase across participants, which meant that the frequency of left-to-right and right-to-left position changes were equivalent for a given exemplar across participants for mismatch probe displays. Within participants, half of the trials associated with a particular scene (e.g., the beach scene) were mismatch trials; for these trials, the critical item was displaced to the left and to the right equally often.

#### Statistical analyses

***Match/Mismatch Decisions***. Discriminability (d') scores and corrected recognition scores were calculated to evaluate match/mismatch response accuracy for each participant. Corrected recognition scores were calculated using the following formula: (Hit Rate + Correct Rejection Rate)/2. Here, hits correspond to manipulated scenes correctly identified as “mismatches” and correct rejections correspond to repeated scenes correctly identified as “matches.” The hit rate (or correct rejection rate) was calculated by dividing the number of hits (or correct rejections) by the total number of hits and misses (or correct rejections and false alarms) for each participant.

After the corrected recognition scores were calculated, an arcsine transformation was applied to the data—this approach was used to circumvent potential violations of the homogeneity of variance assumption that may occur when binary data are summarized as proportions. To improve the equality of variance, extreme values were transformed using the following equations: 1/(4*n*) and (*n* − 1/4)/*n*, for proportions of zero and one, respectively. In these equations, n corresponds to the total number of trials that were used to calculate proportions before the arcsine transformation was applied.

***Change Specification***. Analyses based on change specification were limited to mismatch trials—i.e., trials on which the probe scene was a manipulated version of the studied exemplar. Accuracy on the test of change specification, calculated as the proportion of trials on which participants correctly identified the manipulated item, was evaluated separately as a function of match/mismatch response accuracy. Data were arcsine transformed, as above, before statistical tests were performed.

***Anatomical Considerations***. Because our sample was small, statistical comparisons could not be performed based on differences in the extent and/or location of brain damage. However, the individual performances of each patient are plotted in every figure, and the performances of three patients with noteworthy anatomical profiles are highlighted for purposes of qualitative comparison. One of these individuals (2144, in yellow) has damage limited to the hippocampus, another (1606, in orange) has slight (relative to hippocampus), but significant, gray and white matter volume reductions in the parahippocampal gyrus, and the final individual (2363, in green) has significant gray matter volume reduction in the parietal lobe.

### Results and discussion

**Figure 3:**
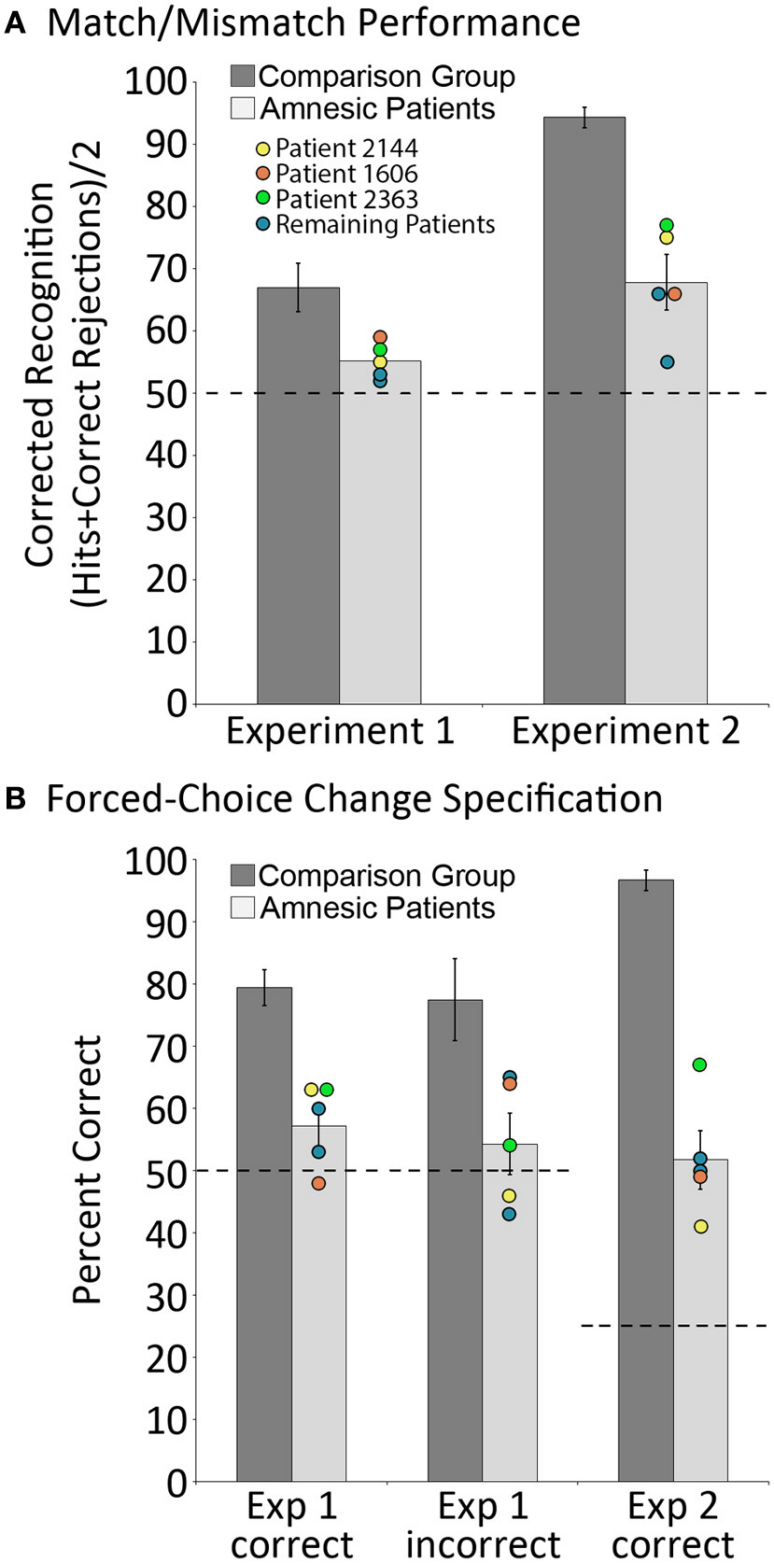
**Behavioral results. (A)** corrected recognition on the match/mismatch test in Experiments 1 and 2, for amnesic patients (light gray bars) and comparison participants (dark gray bars); **(B)** proportion correct on the change specification test in Experiments 1 and 2, contingent on whether mismatching scenes were successfully identified (“Correct”) or not (“Incorrect”), for amnesic patients (light gray bars) and comparison participants (dark gray bars). Individual performances for each patient are indicated, and the performances of three patients with noteworthy anatomical profiles—one with damage limited to hippocampus (2144, yellow) and two with more extensive damage that includes either the parahippocampal region (1606, orange) or the parietal lobe (2363, green)—have been highlighted. Error bars represent standard error of the mean. Dashed lines represent chance level performance.

Relative to matched comparison participants, it was predicted that patients would perform more poorly when they were asked to indicate whether or not the probe stimulus was an exact match of the sample. We were also interested in assessing whether the performance of comparison participants on match/mismatch decisions would be affected (i.e., improved or made worse) by repeated exposure to exemplars of the same scene across trials due either to improvements in scene/object memory, or interference from previous trials. To assess these predictions, a between-groups repeated measures ANOVA with the factors group (amnesic patients, comparison participants) and trial number (trial 1, trial 2 … trial 8) was calculated based on match/mismatch responses. As predicted, comparison participants outperformed amnesic patients on the test of working memory for relationships among items embedded in scene contexts [means (*SD*s) = 66.99 (10.28) and 55.16 (3.11) percent correct, respectively; d' scores = 1.03 and 0.30; *F*'s_(1,11)_ ≥ 5.65, *p*'s < 0.05]. The performances of both groups were above chance [i.e., 50% correct; *t*'s_(4)_ ≥ 3.70, *p*'s ≤ 0.01 and *t*'s_(7)_ ≥ 4.45, *p*'s = 0.001, for amnesic patients and comparison participants, respectively], though the lower bound of the 95% confidence interval for patients was just 51.3% correct (as compared to 58.4% correct for comparison participants). Performance did not change across trials [main effect of trial: *F*'s_(7,77)_ ≤ 1.64, *p*'s > 0.05 for corrected recognition and d' scores] and there was not a significant group by trial interaction [*F*'s_(7,77)_ ≤ 1.22, *p*'s > 0.05 for corrected recognition and d' scores]. The absence of a statistically reliable difference across trials implies that performance was not unduly affected by exposure to several variants of the same scene over the course of an experimental block. The main effect of group, collapsed across trials, is illustrated in Figure 3A. To ensure that this effect was not driven by a longer delay for patients than comparison participants (e.g., because patients were more likely to read, as an instructional reminder, the memory question posed at the bottom of the screen; see Figure 2), the amount of time required to make match/mismatch decisions was calculated for each group. The small between-groups difference in response time (patients: 5.75 ± 1.46 s; comparison group: 5.49 ± 0.82 s) was not statistically reliable [*t*_(11)_ = 0.40, *p* > 0.6].

In addition to the global match/mismatch impairment, comparison participants were expected to outperform patients on the change specification test. Consistent with this prediction, results indicated that amnesic patients performed more poorly than comparison participants when they attempted to identify the item that had been displaced (from two alternatives) when probe scenes were manipulated. When manipulated scenes were correctly endorsed as *mismatches*, comparison participants successfully identified the item that had changed locations 79.46 (*SD* = 7.72) percent of the time, whereas amnesic patients identified that item just 57.19 (*SD* = 6.84) percent of the time. These differences were statistically reliable [*t*_(11)_ = 4.75, *p* = 0.001], and while performances of both groups were above chance [*t*_(7)_ = 8.84, *p* < 0.001 and *t*_(4)_ = 2.35, *p* < 0.05 for controls and patients, respectively], the lower bound of the 95% confidence interval for patients was just 48.7% correct (as compared to 73% correct for comparison participants). Notably, comparison participants successfully identified items that had changed locations 77.49 (*SD* = 17.47) percent of the time even when they had incorrectly endorsed manipulated scenes as *matches*; this change specification rate was reliably greater than chance [*t*_(7)_ = 4.34, *p* < 0.005], and was as good as change specification performance when they correctly endorsed manipulated scenes as *mismatches* [*t*_(7)_ = 0.52, *p* > 0.05]. The same could not be said for amnesic patients, as they identified items that had changed positions just 54.28 (*SD* = 9.91) percent of the time when they had endorsed manipulated scenes incorrectly as *matches*. For patients, change specification was not reliably different from chance [*t*_(4)_ = 0.97, *p* > 0.05], and the between groups performance difference was statistically reliable [*t*_(11)_ = 2.67, *p* < 0.05; see Figure 3B].

Altogether, results from Experiment 1 confirm predicted relational memory impairments in amnesia, and indicate that the difference in performance across groups was better captured by the more sensitive forced-choice measure: comparison participants demonstrated knowledge about the change even when they failed to report it on the match/mismatch test, while amnesic patients performed near chance levels on the change specification test even when they correctly identified manipulated scenes as *mismatches*. To address questions about whether or not the reported outcomes were influenced by repeated testing of three patients (who completed the experiment twice to fill out the counterbalancing), the data were reanalyzed after the performances of these individuals from the second testing session were omitted. As can be seen in the supplementary results, reported outcomes remain the same, thus, eliminating any potential concern about influences of repeated exposure to the experiment on task performance.

## Experiment 2

The use of eight critical items in Experiment 1 made the task challenging even for comparison participants, and may have exceeded the capacity of working memory, which is a criticism that was raised in response to our previous work (cf. Jeneson and Squire, 2011). Therefore, in Experiment 2 the number of critical items embedded in each scene was reduced to four, which is within the capacity limits of working memory (e.g., Luck and Vogel, 1997). Poor performance in Experiment 1 may also have been a consequence of failure to direct attention to critical items when scenes were presented during the sample phase. To address this concern, rather than use orienting questions, critical items were highlighted briefly during the study exposure of the current investigation to ensure that participants attended to them. As in Experiment 1, critical items were the only items that might be subject to a position change when the probe stimulus was presented.

### Methods

#### Participants

Participants were as described above—the same patients and comparison participants completed this experiment, and gave their consent before testing was initiated.

#### Materials

**Figure 4:**
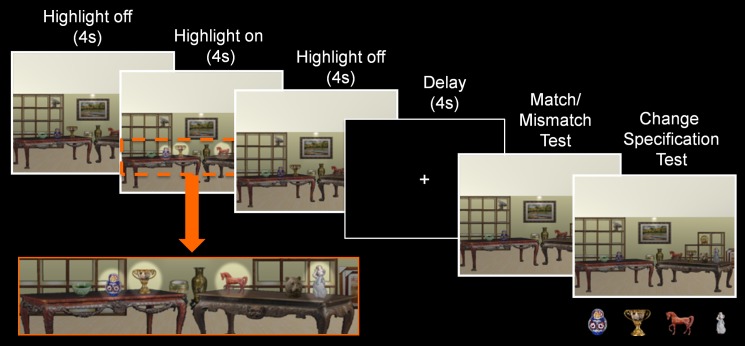
**Trial structure of Experiment 2**. During the sample phase, a scene exemplar was shown in its original format (4 s), followed by a highlighted view of the critical objects (4 s), and a return to its original format (4 s). Subsequent to the delay, a probe stimulus was presented and participants made a match/mismatch decision and attempted to identify the item that had changed locations. Here, pictures of the critical items were presented below the probe stimulus when the change specification test was administered.

A novel set of eight rendered scenes, each with eight corresponding exemplars, were created for Experiment 2 using Punch! Home Design software^©^. The materials were subject to the same development process that was described above for Experiment 1. As in that study, the final set of materials included 128 scenes—eight distinct scene contexts, each with eight different exemplars, and one additional manipulated version of each exemplar (see Figure 4 for an example of one such scene).

#### Design and procedure

Aside from two notable differences, the design used here was identical to the one that was described earlier. Changes to the experimental design included: (1) a reduction in the number of critical items embedded in each scene from eight to four, and (2) a highlighted view of the critical items for 4 s in the middle of the sample phase, which was accomplished by increasing the brightness and contrast of the region where each critical item was located (see Figure 4).

The instructions provided prior to testing were also as described above for Experiment 1, but now participants were told to pay close attention to the items that were highlighted when scenes were presented at the beginning of each trial. The trial structure was modified slightly—the duration of the sample phase was increased to 12 s, a fixation screen was presented during the delay (because the use of a visual mask in Experiment 1 might have contributed to poor performance), and participants made a four-alternative forced-choice change specification decision following the match/mismatch response during the probe phase. In this case, pictures (rather than names) of *all four* critical items were presented at the bottom of the screen below the probe stimulus on every single test trial and participants attempted to identify the item that may have changed locations relative to the sample stimulus. As was the case in Experiment 1, change specification responses were made on every trial irrespective of match/mismatch responses. Three patients (1606, 1846, and 2363) completed the experiment twice with different counterbalanced versions of the task. For these individuals, the experiments were conducted in separate sessions that were scheduled several months apart. As was done for Experiment 1, analyses were based on the averaged data from both sessions for those individuals who completed two rounds of testing. Counterbalancing was also as described above, and debriefing was provided at the conclusion of the experiment.

### Results and discussion

Results replicated those reported for Experiment 1. Between-groups repeated measures ANOVAs with the factors group and trial number, calculated using corrected recognition and d' scores, confirmed that patients performed more poorly than comparison participants when match/mismatch responses were made [*F*'s_(1,11)_ ≥ 52.09, *p*'s < 0.001]. On average, patients and comparisons participants successfully distinguished matching from mismatching probes 67.81 (*SD* = 8.97) and 94.34 (*SD* = 4.49) percent of the time (d' scores were 1.32 and 3.62 for patients and comparison participants, respectively), and performances of both groups were reliably above chance [*t*'s_(4)_ ≥ 4.31, *p*'s < 0.01 and *t*'s_(7)_ ≥ 14.17, *p*'s < 0.001 for patients and controls, respectively; see Figure 3A]. There were no differences in performance across trials [*F*'s_(7,77)_ ≤ 0.88, *p*'s > 0.05], nor was there a statistically reliable group by trial interaction [*F*'s_(7,77)_ ≤ 1.59, *p*'s > 0.05]—both outcomes suggest that performance was not affected by repeated exposure to the same scene context over the course of an experimental block. In addition, the time required to make match/mismatch decisions was comparable for patients and comparison participants [4.91 ± 1.89 and 4.31 ± 0.96 s, respectively; *t*_(11)_ = 0.77, *p* > 0.4].

Consistent with results reported in Experiment 1, comparison participants outperformed patients on the change specification test. Even when patients endorsed manipulated scenes correctly as *mismatches*, they identified the critical object that had been displaced just 51.74 (*SD* = 9.42) percent of the time. This was well below the performance of comparison group participants, who identified the displaced object 96.70 (*SD* = 4.37) percent of the time [*t*_(11)_ = 11.04, *p* < 0.001], but the performances of both groups were reliable greater than chance [here 25% correct, *t*_(4)_ = 6.54, *p* = 0.001 and *t*_(7)_ = 26.30, *p* < 0.001, for patients and comparison participants, respectively; see Figure 3B]. Change specification performance could not be evaluated for comparison participants when manipulated scenes were incorrectly endorsed as *matches* because there were too few trials (i.e., seven errors across eight participants). Among amnesic patients, displaced items were successfully identified 27.99 (*SD* = 19.23) percent of the time following incorrect endorsement of manipulated scenes as *matches*, a score that was not reliably greater than chance [*t*_(3)_ = 0.19, *p* > 0.05 ^1^], and indicates that patients could not identify items that had changed locations following an incorrect match/mismatch response despite the use of a more sensitive forced-choice measure.

Considered together, results from this study replicate and extend those from Experiment 1. Despite a reduction in the number of critical items, and the use of highlighting to ensure that participants attended to these items, the performances of amnesic patients on match/mismatch judgment continued to be severely compromised relative to performances of comparison participants. Perhaps more striking is the severe impairment on the change specification test. Even after having successfully identified a probe scene as a mismatch, amnesic patients only managed to identify the item that had changed locations 52% of the time. Comparison participants, on the other hand, performed near ceiling, identifying the correct item on 97% of correctly endorsed mismatch trials. As was the case for Experiment 1, results remain the same even when the performances of patients who completed the experiment a second time (to fill out counterbalancing) were excluded from the data set (see supplementary results).

## General discussion

In two experiments, the performances of amnesic patients and matched comparison participants were compared on tests that required short-term memory for relationships among items embedded in scenes. The major finding was that despite a short delay of just 4 s, and the absence of intervening items between corresponding study and test displays (as in Hannula et al., 2006), amnesic patients were severely impaired. Impaired performance was evident even when critical items were highlighted during the sample phase so that we could be sure they were attended, and impairments were comparable whether damage was limited to the hippocampus or more extensive (see Figure 3). The absence of any systematic difference in the performances of individual patients is critical, and indicates that reported deficits are not a consequence of, or even exacerbated by, the presence of extra-hippocampal damage. More generally, the results are striking because impairments documented in previous studies that have examined short-term retention of inter-item relationships in amnesia have typically been modest (e.g., Ryan and Cohen, 2004; Hannula et al., 2006; although see Watson et al., 2013), or have not been forthcoming (see Jeneson and Squire, 2011 for review). These disparate outcomes are not easily explained by differences between the patients tested here and elsewhere (Jeneson et al., 2011), as comparisons of basic demographic information (age, education) and performances on standardized neuropsychological tests (FSIQ, GMI; see Table 3) indicate that our patients are no more severely amnesic than others. It seems then, that the short-term memory impairments documented in the current set of studies are a consequence of memory demands imposed by our tasks, though alternative interpretations are also considered in the sections that follow.

**Table 3:** **Demographics and neuropsychological tests scores as compared to patients tested by Jeneson et al. (2011)**.

**Variable of interest**	**Group 1** (**n** = **5**)	**Group 2** (**n** = **5**)	***t*-test**
Age	52.0 (6.3)	56.8 (11.5)	*t*_(8)_ = 0.82, *p* > 0.05
Years of education	14.0 (2.0)	12.3 (0.7)	*t*_(8)_ = 1.80, *p* > 0.05
FSIQ	94.8 (7.3)	101.2 (7.5)	*t*_(8)_ = 1.37, *p* > 0.05
GMI	65.4 (8.8)	72.6 (5.4)	*t*_(8)_ = 0.16, *p* > 0.05
FSIQ minus GMI	29.4 (7.7)	28.6 (9.5)	*t*_(8)_ = 0.15, *p* > 0.05

Group 1, patients tested in the reported experiments; Group 2, patients tested by Jeneson et al. (2011). Standard deviations are presented in parenthesis.

One notable difference between the current experiments and the study conducted by Jeneson et al. (2011) was the omission of orienting questions, which may have elicited strategies that led to compensatory performance advantages when amnesic patients were tested. Our first experiment did not provide participants with any information about which item might subsequently change positions when test displays were presented and it is possible that in the absence of some sort of orienting information, the task drew on long-term memory. In addition, this task was very challenging (comparison group performance was quite poor), which meant that reported impairments may have been a consequence of task difficulty. Therefore, in the second experiment, a subset of items (4 items in a scene context) was highlighted during the sample phase, and it was emphasized to participants that they should pay close attention to these items and their spatial locations. This manipulation was meant to keep processing and representational demands within the capacity limits of short-term memory (e.g., Luck and Vogel, 1997).

Critically, when highlighting was used to encourage processing of a subset of the scene elements, the performance of comparison participants on match/mismatch discrimination decisions improved by 27 percent (i.e., from 67 percent correct in Experiment 1 to 94 percent correct in Experiment 2); corresponding improvements were modest among amnesic patients, whose performances improved by just 13 percent (i.e., from 55 percent correct in Experiment 1 to 68 percent correct in Experiment 2). While we cannot rule out the possibility that having highlighted a subset of items encouraged active verbal rehearsal of to-be-retained object-location relationships, there is no reason to suspect that this strategy would be adopted more often by comparison participants than patients. The patients tested here perform within normal limits on tests of executive function (see Konkel et al., 2008), and showed modest improvements in Experiment 2, which indicates that they understood the import of highlighted objects. Still, they were unable to capitalize on this information to the same extent as controls. Thus, even when load and trial structure made short-term retention strategies more likely, amnesic patients continued to be severely impaired. This outcome renders difficulty-based interpretations of the reported results unlikely because changes in task design that elicited near-ceiling discrimination of repeated from manipulated scenes among healthy comparison participants were not sufficient to rescue patient performance.

Another difference between our work and past studies that have required short-term retention of relationships among item embedded in scenes (Ryan and Cohen, 2004; Hannula et al., 2006; Jeneson et al., 2011), was the decision to use the same limited set of scenes repeatedly across several successive trials. This approach minimized any potential contributions of item or scene memory to task performance, and meant that relational memory could be examined in relative isolation (i.e., performance hinged on flexible encoding and active retention of currently relevant relationships among items). Moreover, active retention of the absolute or metric location of just one or two objects was discouraged because participants could not predict which object might be subject to displacement (as might have been the case when orienting questions were used). That robust impairments were a consequence of requirements to retain active representations of the relative positions of objects embedded in scenes is consistent with results reported by Watson et al. (2013) who demonstrated that the hippocampus is especially important for object-to-*relative*-location binding. In that experiment, patients and controls studied a small array of objects, and following a short delay, were asked to place the objects where they had been located during the sample phase (see also Smith and Milner, 1989; Jeneson et al., 2010). The most common memory error, made nearly 40 times more often among patients than controls, was one in which participants swapped the locations of two objects, which suggests that memory for filled locations was retained, but that the item-to-location or inter-item bindings were not. While the manipulations used here in mismatch displays were not ones in which two items swapped locations, the absence of an orienting question that highlighted a critical item combined with repeated presentations of the same scene contexts may have encouraged participants to rely on the relative locations of objects during the sample phase.

Our results are complemented by past reports that have documented impaired retention of object-location associations in the absence of scenes contexts (Olson et al., 2006; Finke et al., 2008, 2013; Watson et al., 2013). In two of these investigations (Olson et al., 2006; Finke et al., 2008), patients and controls attempted to retain objects (colors), locations, or object-location (color-location) associations over the course of a short delay and associative memory was selectively impaired. Like ours, these findings implicate the hippocampus in short-term retention spatial relationships, but others maintain that the reported impairments reflect unanticipated contributions of long-term memory to performance (e.g., Shrager et al., 2008). To test this hypothesis, a simple method was introduced to determine whether or not performances on tests that nominally require active retention (e.g., based on trial structure) are actually mediated by long-term memory retrieval. The logic was that distraction, introduced during the retention interval of delay-based tasks, should only disrupt performances of healthy comparison participants when a sample stimulus was being actively retained. If performance was unaffected by distraction, then the sample stimulus must have been recovered from long-term memory. By extension, amnesic patients should only be impaired on the subset of “short-term” memory tests that depend on long-term memory retrieval. When this logic was applied to tests that required active retention of object-location associations, control performance was poorer in the presence of distraction whether memory for three (low load) or six (high load) associations was tested, and amnesic patients were impaired in the high-load condition. On its face, this outcome appears to confirm hippocampal contributions to active retention of object-location associations, but because the magnitude of the interference effect was less robust for high- than low-load trials in control data, impaired performance was said to reflect load-based long-term memory dependence. At the very least, this interpretation suggests that the interference method cannot be used to unambiguously determine whether performance is supported by active online retention of studied content, and under these circumstances, the method itself becomes questionable because it is possible to interpret results in a variety of ways despite the presence of interference effects in control group performance. More generally, it is notable that relational memory deficits following hippocampal damage have not always been limited to high-load conditions; impairments were reported by Finke et al. (2008; see also Watson et al., 2013) when individuals with right-lateralized hippocampal lesions were tested, even when memory load was limited to just two associations. Therefore, the confluence of evidence seems to suggest an extended reach of the hippocampus to representation of relationships among items regardless of time scale (see also Voss et al., 2011; Warren et al., 2011, 2012a).

One final notable outcome of the reported studies concerns performance on the change specification test. In experiment 1, performances of participants from both groups indicated that it was difficult to accurately distinguish matching from manipulated pictures presented during the probe phase. However, research has shown that these decisions can be influenced by the willingness of participants to make a particular type of response (i.e., the criterion that is set). In other words, participants may detect changes, but fail to report them because they lack confidence in the accuracy of their memories (see Hannula et al., 2005 for discussion). Therefore, the use of a more sensitive measure, here, change specification, has potential to provide additional insights into whether or not information was successfully retained in memory over the course of a delay. In our case, the decision to include a forced-choice test that required participants to identify the object that might have changed locations whether scenes were endorsed as manipulated or not was fortuitous and unveiled memory for studied relationships in our comparison group that would have otherwise gone undetected. Indeed, even when comparison participants incorrectly indicated no change on the match/mismatch decision, they successfully identified the displaced object 77% of the time. The same could not be said for amnesic patients, who only identified the displaced object 57% of the time when manipulated scenes were endorsed *correctly* as mismatches. This outcome hints at the possibility that amnesic patients were guessing about the status of some scenes when match/mismatch decisions were made, or that the information retained in memory was impoverished and insufficient to support change specification. Poor performances of amnesic patients on the change specification test were evident in Experiment 2 as well. Following correct endorsement of a scene as manipulated, patients identified the item that had been displaced just 52% of the time. This was well below the performance of comparison participants, who identified the displaced item correctly on 97% of the trials. In sum, comparison participants had more precise memory for spatial relationships than was suggested by their match/mismatch performance (Experiment 1) and patients performed poorly on the change specification test even when their match/mismatch decisions were correct (Experiments 1 and 2).

The outcomes described above are consistent with our view that the hippocampus is critically involved in mediating representations of arbitrary relationships among items that themselves are the constituent elements of experiences and events (cf. Cohen and Eichenbaum, 1993; Eichenbaum et al., 1994; Konkel and Cohen, 2009). The results suggest that when task performance depends critically on relational memory representations, and contributions of other types of memory (e.g., memory for individual items, memory for contexts) have been minimized or eliminated, performances of patients with hippocampal damage will be compromised whether memory is tested immediately or after a considerable delay. That said, it is important to consider alternative explanations for the reported outcomes, and we do so now.

One possibility is that compromised performance reflects perceptual processing impairments. This is suggested by recent work that has implicated the hippocampus in successful perceptual discrimination of visually similar complex scenes (cf. Lee et al., 2012). In the absence of a perceptual control condition, the possibility that perceptual deficits contributed to our results cannot be dismissed definitively. However, some leverage against a strong version of the perception-based interpretation is provided by (1) a comparison of our scenes to materials used in studies that have documented perceptual impairments following hippocampal damage, and (2) by findings from our lag-based experiment (Hannula et al., 2006). First, materials used in studies that have documented perceptual impairments have either been morphed versions of scenic pictures, or sparse rendered rooms (i.e., devoid of objects) presented from different viewpoints. In contrast, our scenes were visually rich pictures with several embedded objects, the sample and the probe were always presented from the same perspective, and exemplars of same scene were not morphs of one another, but rather were discriminable based on a change in the spatial location of at least one object. Whether or not this kind of manipulation might elicit impaired perceptual discrimination among hippocampal amnesics remains to be determined, but this seems unlikely, as proponents of the representational-hierarchical model have indicated that successful discrimination is likely to occur when scenes can be distinguished based on the characteristics of individual features or objects (see Lee et al., 2012). The key finding from our past work that argues against compromised perceptual processing is based on the observation that the same patients tested here could successfully respond to orienting questions that required evaluation of the very relationships that might subsequently be manipulated when scenes were presented again later (Hannula et al., 2006). This outcome suggests that patients can process items and inter-item relationships when complex scenes, like the ones used here, are in view. That said, it may be the case that there are some systematic differences in how patients and comparison participants process scenes over the course of a 10 (or 12) second exposure—for instance, comparison participants, but not patients, may spontaneously revisit regions of interest that were not well encoded earlier in viewing (cf. Voss et al., 2011). This possibility could be addressed in future studies, which should include perceptual control conditions so that investigators can more effectively adjudicate between perception- and memory-based accounts of any reported impairments.

A second possibility is that reported results reflect a more general deficit in memory for scenes, though this explanation seems unlikely. While the current set of studies did not examine memory for scenes, our lag-based experiment, which was conducted with the same patients who were tested here, did examine the integrity scene memory (Hannula et al., 2006). In that experiment, memory for relationships among items embedded in scenes was impaired despite successful identification of studied scenes as old. It also seems unlikely that accelerated degradation of scene representations is contributing to the reported outcome, as memory for studied scenes in Hannula et al. (2006) remained quite high even when as many as nine trials separated the initial presentation of a scene from its subsequent reappearance. While we cannot conclusively rule out the possibility that there may be some accelerated degradation of scene representations in our patients—perhaps because they cannot effectively encode/retain detailed information about relationships among items embedded in scenes—it seems unlikely (in light of past work) that accelerated forgetting of scenes is driving the deficit reported here when study and test displays are separated by a very short unfilled delay.

Like several of the investigations cited above in support of hippocampal contributions to short-term memory, the experiments reported here examined memory for *spatial* relationships. This begs the question, are the reported impairments a consequence of spatial processing demands, or can they be attributed more broadly to relational memory requirements? In our own work, we have documented impaired retention of arbitrary scene-face pairings when test trials are presented immediately after corresponding study trials—an effect that cannot be attributed to spatial processing (Hannula et al., 2006). However, a continuous recognition design was used in that study and so some of the same criticisms that were raised in response to our work with items embedded in scenes (Jeneson et al., 2011) also apply to the scene-face investigation. Others have examined memory for color-shape, color-letter, or word–word bindings (Baddeley et al., 2010; Braun et al., 2011) in patients with circumscribed hippocampal damage, and have reported no deficits relative to matched comparison participants. At face value, these outcomes seem to argue against a broader relational memory hypothesis, but we would propose that these feature and word binding tasks do not depend critically on the integrity of the hippocampus. Indeed, as predicted by the relational memory theory (Cohen and Eichenbaum, 1993), neuroimaging and neuropsychological investigations indicate that configural or unitized intra-item and word-word associations can be supported by MTL cortical structures (e.g., perirhinal cortex; e.g., Quamme et al., 2007; Diana et al., 2008; Haskins et al., 2008; Staresina and Davachi, 2008, 2010). These types of bindings lack properties of flexibility and compositionality that are said to characterize relational memory representations (Cohen and Eichenbaum, 1993; see Haskins et al., 2008), and as such it is not surprising that impaired performances were not evident when short-term retention was tested. This explanation for the reported absence of hippocampal-dependence in the above-referenced short-term memory tests is consistent with results from a recent neuropsychological investigation which showed that active retention of configural memory representations (in this case memory for faces) depends critically upon the integrity of MTL cortical structures, but not the hippocampus (Race et al., 2013). Ultimately, it seems that the literature addressing questions about hippocampal contributions to short-term memory has become a bit lopsided in favor of tasks that tax spatial relational memory. Future investigations might therefore address questions about whether or not inter-item, item-context, and temporal relationships can be retained over the short-term by hippocampal amnesic patients.

### Conflict of interest statement

The authors declare that the research was conducted in the absence of any commercial or financial relationships that could be construed as a potential conflict of interest.

## Footnotes

^1^One patient was excluded from this analysis because he did not incorrectly endorse any of the manipulated scenes as matches.
