# Supplementary material for: Short-term retention of relational memory in amnesia revisited: accurate performance depends on hippocampal integrity
Source: Front Hum Neurosci. 2014 Jan 24;8:16. doi: 10.3389/fnhum.2014.00016 (PMC3901041; doi:10.3389/fnhum.2014.00016)
Supplement: Supplementary file 1 [file DataSheet1.PDF]

SUPPLEMENTARY RESULTS

As indicated in the text, three patients were tested twice in Experiment 1 and Experiment 2 to fill out the counterbalanced experimental design. Here, we reanalyze the data, eliminating performances of patients from the second round of testing (along with the performances of matched comparison participants). Note that comparison participants did not complete the experiment twice - instead, when patients were tested twice, two matched comparison participants were recruited to participate in the experiment.

<u>Experiment 1</u>

A between-groups repeated measures ANOVA with the factors group (amnesic patients, comparison participants) and trial number (trial 1, trial 2 … trial 8) was calculated based on match/mismatch responses. As predicted, comparison participants outperformed amnesic patients on the test of working memory for relationships among items embedded in scene contexts (means (SDs) = 67.19 (12.00) and 55.00 (4.19) percent correct, respectively; d’ scores = 1.05 and .25; $F\text{'s}(1,8) \geq 5.93$, $p\text{'s} < .05$). The performance of comparison participants but not amnesics was above chance (i.e., 50% correct; $t\text{'s}(4)=2.67$ and 3.04, $p\text{'s}=.056$ and .04, for amnesic patients and comparison participants, respectively), though the lower bound of the 95% confidence interval for both groups was near chance (49.8% correct for patients and 51.6% correct for comparison participants). Performance did not change across trials (main effect of trial: $F\text{'s}(7,56) \leq 2.48$, $p\text{'s} > .05$ for corrected recognition and d’ scores) and there was not a significant group by trial interaction ($F\text{'s}(7,56) \leq 0.89$, $p\text{'s} > .05$ for corrected recognition and d’ scores). The absence of a statistically reliable difference across trials implies that performance was not unduly affected by exposure to several variants of the same scene over the course of an experimental block.

In addition to the global match/mismatch impairment, comparison participants were expected to outperform patients on the change specification test. Consistent with this prediction, results indicated that amnesic patients performed more poorly than comparison participants when they attempted to identify the item that had been displaced (from two alternatives) when probe scenes were manipulated. When manipulated scenes were correctly endorsed as *mismatches*,

comparison participants successfully identified the item that had changed locations 79.19 (SD=9.19) percent of the time, whereas amnesic patients identified that item just 56.61 (SD=5.66) percent of the time. These differences were statistically reliable (t(8)=4.18, p=.003). The performance of comparison participants but not amnesics was above chance (t’s(4)=5.81 and 2.61, p’s=.004 and .059 for comparison participants and patients, respectively). Notably, comparison participants successfully identified items that had changed locations 81.92 (SD=16.70) percent of the time even when they had incorrectly endorsed manipulated scenes as *matches*; this change specification rate was reliably greater than chance (t(4)=4.13, p=.014), and was as good as change specification performance when they correctly endorsed manipulated scenes as *mismatches* (t(4)=.20, p>.05). The same could not be said for amnesic patients, as they identified items that had changed positions just 54.46 (SD=8.03) percent of the time when they had endorsed manipulated scenes incorrectly as *matches*. For patients, change specification was not reliably different from chance (t(4)=1.25, p>.05), and the between groups performance difference was statistically reliable (t(8)=3.27, p=.011).

<u>Experiment 2</u>

Results replicated those reported for Experiment 1. Between-groups repeated measures ANOVAs with the factors group and trial number, calculated using corrected recognition and d’ scores, confirmed that patients performed more poorly than comparison participants when match/mismatch responses were made (F’s(1,6)≥33.91, p=.001). On average, patients and comparisons participants successfully distinguished matching from mismatching probes 64.30 (SD=11.48) and 95.41 (SD=3.51) percent of the time (d’ scores were 1.09 and 3.79 for patients and comparison participants, respectively), and performances of both groups were reliably above chance (t’s(4)=2.74 and 17.32, p=.05 and p<.001, respectively). There were no differences in performance across trials (F’s(7,42)≤.39, p’s>.05), nor was there a statistically reliable group by trial interaction (F’s(7,42)≤1.80, p’s>.05) – both outcomes suggest that performance was not affected by repeated exposure to the same scene context over the course of an experimental block.

Consistent with results reported in Experiment 1, comparison participants outperformed patients on the change specification test. Even when patients endorsed manipulated scenes correctly as

mismatches, they identified the critical object that had been displaced just 50.53 (SD=11.85) percent of the time. This was well below the performance of comparison group participants, who identified the displaced object 98.63 (SD=1.88) percent of the time (t(8)=11.06, p<.001), but the performances of both groups were reliable greater than chance (here 25% correct, t's(4)= 4.97 and 38.43, p's<.01, for patients and comparison participants, respectively). Change specification performance could not be evaluated for comparison participants when manipulated scenes were incorrectly endorsed as *matches* because there were too few trials (i.e., five errors across five participants). Among amnesic patients, displaced items were successfully identified 28.21 (SD=19.60) percent of the time following incorrect endorsement of manipulated scenes as *matches*, a score that was not reliably greater than chance (t(3)=.05, p>.051)[1], and indicates that patients could not identify items that had changed locations following an incorrect match/mismatch response despite the use of a more sensitive forced-choice measure.

---

[1] One patient was excluded from this analysis because he did not incorrectly endorse any of the manipulated scenes as *matches*.
